# Experiences of Newly Qualified Nurses’ Engagement with Quality Improvement in Practice: A Qualitative Follow-Up Study

**DOI:** 10.3390/nursrep14040218

**Published:** 2024-10-14

**Authors:** Lorraine Armstrong, Ashley Shepherd, Fiona Harris

**Affiliations:** Faculty of Health Sciences, University of Stirling, Stirling FK9 4LA, UK; ashley.shepherd@stir.ac.uk (A.S.); fiona.harris@stir.ac.uk (F.H.)

**Keywords:** quality improvement, nursing students, degree-level education, practice, qualitative analysis, follow-up, nurses, engagement, lived experience

## Abstract

Background: Nurse education plays an essential role in preparing future nurses to engage with quality improvement (QI) initiatives in their organisations and improve patient care. However, frontline nurses continue to report that a lack of QI knowledge hinders their abilities to engage in improvement work. In the UK, student nurses are now trained in QI within their degree to enable them to contribute to improvements once qualified. Objectives: This qualitative follow-up study investigated the sustainability of QI engagement in nurses who undertook QI training and a QI project during their degree and explored the factors influencing their engagement in QI once qualified. Methods: This paper followed the COREQ criteria to report upon 10 semi-structured interviews undertaken with registered nurses and focuses on their experiences of QI engagement post-registration. The data were investigated using an inductive thematic analysis and Nvivo 14. Findings: Five themes emerged: transition to a newly qualified nurse, QI knowledge decline, influencing factors (hierarchy, leadership, COVID-19 pandemic, data access and location), and skill transferability. Conclusions: This study showed that qualified nurses can sustain their QI knowledge and remain engaged with QI where they experience positive QI leadership and were exposed to ongoing QI activity in their preceptorship year. However, a lack of QI opportunities and a culture which does not consider QI a responsibility of new nurses is seen to hinder engagement. Educational institutions and practice partners require careful collaboration to assess and develop ongoing QI learning activities that support new nurses to engage in QI.

## 1. Introduction

There is a drive in the UK (United Kingdom) and internationally to increase quality and service improvement content within nursing degree programmes to prepare future nurses to improve health and care services. This initiative is in response to professional standards set out by the Nursing and Midwifery Council (NMC) who make safety and quality of care an explicit proficiency to achieve [1]. To help inform faculties on how to develop and evaluate quality improvement (QI) curricular content in their programmes, a collection of research reviews and international primary research articles have focused on exploring QI in degree level nurse education [2,3,4,5,6]. This evidence confirms that student nurses learn to contribute to systematic change through practice-based QI projects and there is compelling evidence to suggest this pedagogical approach, in the right context, can foster QI-related behaviour change [7].

In 2016, a Scottish university in partnership with their local health board serving a population of around 300,000, facilitated a QI practice-based educational assessment called the QI Practicum. The development phase and QI intervention are described elsewhere [8] and detailed in Table 1.

The QI Practicum was designed to enable student nurses to gain practical experience of QI during an 8-week placement and identify and test changes to improve an area of practice using an established QI model. Powell et al. [9] report that the five most common improvement models used in healthcare are total quality management or continuous quality improvement, business process reengineering, lean thinking, six sigma, and IHI’s rapid cycle change, also referred to as PDSA cycles. In the QI Practicum, students were taught about PDSA cycles as part of the model for improvement through seminars, e-learning modules, webinars, and podcasts [10].

This model suited the students’ level of education and offered a ‘bottom-up’ approach for them to find problems and test low risk solutions in practice [10]. In line with other studies, the students used QI tools during their projects which included process mapping, pareto charts, driver diagrams, run charts, cause/effect diagrams, and bar graphs [8,11,12]. Students completed QI projects in pre-operative assessment, community health centres, hospitals, hospices, nursing homes, the emergency department, and acute care. Areas they focused on to improve included oral care, handover communication, the inclusion of patients’ carers, adherence with personal protective equipment, reducing pressure damage and creating dementia friendly environments. A previous teaching evaluation of the QI Practicum concluded that where students engaged in QI in the practice setting there was a transformation from panic and an ignorance of QI to a state of appreciation of and commitment to using QI methods in the future [8].

Following their three-year degree programme, students registered as newly qualified nurses (NQNs) with the UK NMC [13]. Some NQNs secured posts locally in their health boards or private organisations, others returned to their home countries to practice, and some worked in different health boards or practiced internationally. To develop into confident and capable registered practitioners, those who remained in Scotland undertook a national development programme called Flying Start within their first 12 months [14]. This transition programme is designed to combine individual learning with support in the workplace to help NQNs gather evidence and demonstrate that they are upholding professional standards. Thereafter, they join frontline nurses in building evidence portfolios to support their professional development and prepare for a revalidation process every three years to maintain their NMC registration. Despite professional standards making safety and quality of care an explicit proficiency to achieve, there is consensus that a knowledge gap remains as to whether student nurses who received degree level QI education continue to be engaged in QI once qualified. This study therefore aimed to explore the sustainability of QI engagement in post-registration nurses and identify the factors influencing their engagement.

### 1.1. QI Education

The international literature offers various definitions of QI. Batalden and Davidoff [15] present a holistic overview of QI and propose it is ‘the combined and unceasing efforts of everyone—healthcare professionals, patients and their families, researchers, payers, planners, and educators—to make the changes that will lead to better patient outcomes (health), better system performance (care) and better professional development (learning)’. Cepero [16], on the other hand, defines the method as a practical and concise approach that involves ‘a cyclical process designed to evaluate workflow and clinical indicators or outcomes’. It is reasonable to suggest that QI is in fact an umbrella term that encompasses multiple systematic ‘change methods’ to support improvement and better outcomes for patients and services [10,17].

The UK pre-registration nursing workforce is expected to initiate improvements and demonstrate QI competence prior to registration [13]. Over the years, student nurses have been taught QI concepts such as improvement methods, the model for improvement, quality indicator measures, plan–do–study–act (PDSA) cycles, root cause analysis, systems thinking, interprofessional learning, clinical governance, data, human factors, and evidence-based practice [8,11,18]. Approaches to teaching QI have ranged from inviting QI experts to talk with students to involving them in clinical audit and completing QI projects [5]. The latter pedagogical approach is known to foster experiential learning and supports student nurses to apply the theoretical concepts in the real world and develop their QI competencies [2]. By teaching QI this way, it is suggested that organisations benefit from student nurses who can offer added capacity and support to implement improvement projects [19]. Student nurses also gain a sense of belonging in situations where they contribute to policy and protocol changes in real-time while learning from the interprofessional team [3].

However, QI education is complex and comprises components relating to the faculty, the learner, the practice setting, the professional team, the patient, and the QI endeavour. The practice settings in which nurses work are equally complex and involve the physical space, psychosocial and interaction factors, and organizational culture [20,21]. The inter-relationships occurring between these factors is what makes it difficult to identify causality between QI educational assessments and nurses’ future engagement with QI. However, many studies have attempted to explore nurses’ QI engagement.

### 1.2. QI Nurse Engagement

There has been a shortage of attempts to define QI engagement in the nursing literature. For the purpose of this paper, we defined nurse engagement in QI as undertaking QI training after qualifying, involvement in QI initiatives, or applying QI methods/tools to projects in the practice setting.

A systematic review finds that studies reporting on the engagement of nurses in QI have been published for almost 20 years [22]. The findings of these studies show that where nurses engage in QI, improvements have been seen in patient outcomes relating to falls (23%), blood stream infections (70%), and pressure damage (66%) as well as job satisfaction for nurses and reduced staff sickness. A substantial proportion of the healthcare workforce is made up of frontline staff nurses who are ideally placed to identify opportunities for improvement. However, a recent survey (*n* = 409) showed that this population perceived themselves to have low levels of preparedness in quality and safety and showed that two thirds of frontline nurses were not currently involved in any QI or patient safety initiatives [23]. These findings are consistent with the results determined by a ‘QI in practice tool’, which aimed to determine nurses’ QI engagement (*n* = 681). The findings showed that a third of nurses were unsure whether their unit had conducted QI and less than 50% reported taking part in QI [24].

A recent qualitative study which explored nurses’ QI engagement emphasised that leadership influence on QI culture such as creating buy-in, supporting a just culture, and working in partnership with frontline nurses is essential [25]. Nurse leadership involvement has been known to influence the success of QI for over two decades and they currently remain the most engaged subgroup of nurses in QI [23,24,26]. A nurse leaders’ role in engaging frontline nurses is crucial to overcome the added organisational barriers that nurses face such as time pressures, lack of QI expertise, siloed departments, and access to prompt data [25]. These barriers are unlikely to be overcome if nurses are not adequately trained in the foundations of QI. Given that only 33% of early career nurse managers felt prepared by their employers to use QI data analysis tools, the workforce cannot rely upon leaders alone to increase the levels of frontline nurses’ engagement with QI [27]. Earlier research concludes that there is value in determining whether nurses who completed QI training and a QI project during their degree programme remain engaged with QI as qualified practitioners [6,8,22,23]. So, this was the aim of our study.

### 1.3. Context

The study reported in this paper includes the second and final phase of a longitudinal evaluation of the QI Practicum.

The first phase was undertaken between 2016–2018 with the purpose of exploring what contextual factors influenced students’ QI learning experiences in the practice setting. Ethnographic methodology was employed to capture the students’ complex and everyday lived experiences within the cultural context of their practice placements. Data collection included 84 h of participant observations with 30 adult field student nurses across nine clinical practice settings within an acute hospital. Fieldwork occurred over two months using informal interviewing, in-depth interviews, focus groups, and documentary analysis. This methodology enabled new insights into QI in nursing education where previous studies’ designs such as pre-/post-surveys or self-reported data have limited understanding [2]. Discussing the findings from phase one is outside of the scope of this paper. However, in sum, student nurses’ QI learning experiences were influenced by sixteen contextual factors grouped into five themes: practice learning environment, organisational culture, leadership, data for improvement, the assignment, and the student nurse. These factors were experienced as facilitators or barriers, depending on the students’ practice setting. Further, the inter-relationship of contextual factors differed between students and influenced the level on which they engaged in their QI Practicums, ranging from no engagement to engaging in organisational projects. The findings are significant in that they provide new perspectives of undertaking QI education in practice for pre-registration nurses and can inform academic and practice partners who are integrating and/or supporting QI education. Phase two of the study is now reported.

The second and final phase of the evaluation, which is reported in this paper, involved follow-up interviews with the same study participants once they qualified as registered nurses. The purpose was to determine if they had remained engaged with QI as qualified nurses and determine what the factors were influencing their engagement.

## 2. Methods

### 2.1. Design

Phase two of the study used qualitative semi-structured follow-up interviews, and an inductive thematic analysis [28]. Qualitative research is described as ‘an approach for exploring and understanding the meaning individuals or groups ascribe to a social or human problem’ (p. 4) [29]. We considered this design to be the most appropriate to explore phenomena not yet thoroughly researched, such as newly qualified nurses’ long-term engagement with QI. Corben and Strauss [30] refer to qualitative research as dynamic, and we believe this fits with the principles that underpin improving complex and fluid healthcare. The literature has previously criticised designs using pre-/post-surveys and self-reported data in QI educational evaluations [2]. However, our study does not use qualitative interviewing in isolation to explore nurses’ QI experiences. Instead, semi-structured interviews are used to supplement a larger ethnographic evaluation. Due to the disparity in nurses’ work locations in phase two, a multi-site ethnography was not appropriate. To ensure explicit and comprehensive reporting, we applied the consolidated criteria for reporting qualitative research (COREQ) checklist [31].

### 2.2. Research Team

The research team consisted of three females. The lead researcher was a nurse and PhD candidate who held positions during the first and second phase of the evaluation as a clinical academic whose focus was on QI and then a lecturer in nursing (LA). The two supervisory team members included a Professor of Nursing with QI subject expertise (AS) and a non-clinical Professor of Social Anthropology (FH) without QI knowledge but had extensive qualitative training and experience. While the research team were resident at the same university, none of the research team were involved in delivering teaching or marking the QI Practicum for the participants involved in this study.

### 2.3. Participants

As the intention of this study was to follow up on previous participants who had undertaken the QI Practicum to determine their engagement with QI once qualified; a purposive sample was used initially. Participants (n = 30) were approached through their email addresses and/or telephone numbers provided during the first phase of the evaluation; however, some contact details were no longer valid. Where responses were not received, a second attempt to contact participants was made and a message left on their voicemail. A final attempt to reach participants was made through searching public social media pages where contact was made through their private messaging option. A total of 17 participants responded and received follow-up study information sheets by email. They were given seven days to have queries answered and decide whether to take part in the study. Although our original ethics approval permitted us to make contact with participants in a follow-up study, and participants had consented to be contacted at any time, we re-established consent before interviews took place. Consent was recorded verbally at the start of each interview. Overall, seven participants decided not take part in an interview due to being on maternity leave (n = 3), expressing a lack of time through work commitments (n = 2), or did not attend the interview (n = 2). Participants were contacted again to reschedule where they did not attend but no reply was received by the research team. Therefore, the inclusion of participants in the study was reduced to a convenience sample (n = 10).

The mean age of participants was 38 (range 29–47) and the group consisted of 8 females and 2 males. Included in the sample were front-line staff nurses (n = 6), senior staff nurses (charges *n* = 3), and a former staff nurse who had recently left the profession (n = 1). The sample reflected the variety of engagement witnessed during their QI Practicums. Study sample characteristics can be found in Table 2. While we acknowledge that demographic characteristics can influence nurses’ attitudes towards QI, such as salary and working in private or public service [32], the analysis of such was outside of the scope of this study and was not included.

### 2.4. Data Collection

Data collection was undertaken between February and April 2024 by the lead researcher (L.A.). To enable objectivity during data collection, a second member of the supervisory team was present at the start of data collection to oversee the conduct of the first few interviews and to be able to contribute to refining future iterations of the interview schedule (F.H.). A combination of face-to face interviews online through Microsoft Teams (n = 5) and telephone interviews (n = 5) were undertaken. A semi-structured qualitative interview guide was designed and informed by the analysis of contextual factors influencing students’ engagement in the QI Practicum during the first phase of the evaluation. A key focus of the follow-up interviews was to explore whether QI knowledge had been sustained and to explore the nuances involved with QI engagement once qualified and within nurses’ current practice. Given the timeframe between the first and second phase of the evaluation, strategies to mitigate potential participant recall were undertaken. A preliminary meeting was held before each interview where the researcher provided a summary of the QI practicum and an account of the individuals’ reported experiences of their QI engagement at that time. Participants were given an opportunity to reflect and ask questions before they participated in the interview. The interview guide was piloted with one nurse initially and thereafter reviewed by the research team through regular discussion. The interview guide was adjusted accordingly to reflect the analysis during phase one, address the current study aims and account for any new and emerging ideas (Supplementary Materials File S1). Interviews lasted approximately 30 min and were audio-recorded and automatically transcribed using Microsoft Teams (https://www.microsoft.com/en-us/microsoft-teams, accessed on 1 February 2024). Recurring themes were identified with no new data emerging following eight interviews. Two further interviews were conducted to help validate findings. The lead researcher (L.A.) reviewed all transcripts for accuracy of text and corrected anomalies produced by Microsoft Teams. Each supervisory member of the team cross-checked 10 percent of transcriptions for accuracy (F.H./A.S.).

### 2.5. Qualitative Analysis

Our thematic analysis of interview transcripts was guided by Corbin and Strauss’ analytic strategies [30] and managed through Nvivo qualitative data analysis (CDAS), version 14.23.0 (Lumivero, Denver, CO, USA). To become acquainted with the data, transcripts were read in-depth by the lead researcher (L.A.). The supervisory team each read twenty percent of the transcripts (A.S./F.H.). Initial themes were identified in advance using the coding framework generated within the first phase of the evaluation to search for similarities and differences in the data relating to factors influencing nurses’ QI engagement once qualified (Supplementary Materials File S2). This original coding framework from phase 1 had been developed and validated by all of the research team (L.A., A.S., and F.H.). To remain open to new ideas and themes within the data, interview scripts were coded individually, firstly by the lead researcher (L.A.). To mitigate bias, each member of the supervisory team thereafter coded twenty percent of the transcripts to check for inter-rater reliability. New codes and ideas were explored by the researcher team during a debriefing session where probing questions emerged from QI and non-QI perspectives to justify new and emerging ideas. A constant comparison approach between participants’ experiences was then undertaken until no new codes were identified. Themes and subthemes were reviewed and finalised by the lead researcher (L.A.) and agreed by the research team [30].

### 2.6. Ethical Considerations

The University of Stirling Research Ethics Committee (SREC) approved the study on the 7 December 2015 (Ref: SREC 15/16—Paper No. 40). The research study was underpinned by the principles within the Data Protection Act [33] and conducted per the University of Stirling Code of Good Research Practice and NHS (National Health Service) Code of Confidentiality [34].

## 3. Results

### 3.1. Transition to Newly Qualified Nurse

The QI Practicum had intended to equip the next generation of nurses with the QI knowledge and skills required by the NMC. Some NQNs reported placing a value on undertaking QI and included their skills and experiences in their job applications and curriculum vitae (CVs). Some had drawn upon their QI experience during job interviews, which led to one NQN being offered a job in the ward she had completed her QI project in:

*‘I put that in my CV and then discussed it at length with the Charge Nurse at my interview, because she obviously helped me implement it. We kept it running in the ward and when I started there, I used it for my flying start and became one of the link nurses for QI.’*

(Interview D)

Other NQNs did not contemplate adding QI skills to their CVs or job applications. Further, these nurses reported that managers in their preceptorship year asked them to focus on learning the basic fundamental skills of a NQN for at least 6–8 months prior to increasing their responsibilities. This manager advice aligned to students’ beliefs that QI felt like an unnecessary extra as well as being an unsuitable time to be involved during their first year:

*‘Immediately after I qualified my first goal was to actually get more experience because you don’t really know anything. So, my first goal was to learn my skills, and just be enough. I don’t think my focus was on improvement.’*

(Interview A)

### 3.2. QI Knowledge Decline

Beyond preceptorship, the nurses discussed QI-related behaviours but demonstrated less knowledge. They spoke of writing reflective accounts about QI during their NMC revalidation and being involved in improving admissions paperwork, enhancing patient transition to short stay wards, and creating sustainable healthcare delivery through single-use medication cups. Despite associating their behaviours with improvement, the nurses admitted to using a less formal QI process:

*‘The basics of QI are straightforward, you do it without thinking about it—it’s just that I’ve not necessarily used all the diagrams and everything, there were no charts that we used.’*

(Interview E)

*‘I have engaged in improvement, but not in a proper assessed way you know, it doesn’t go through a process, but it’s just me wanting to work better to improve.’*

(Interview A)

Although nurses were taught the model for improvement during their degree, their recall of QI knowledge and terminology during interviews was vague. They admitted revisiting old coursework prior to their interview to remind themselves of the QI process. A ‘use it or lose it’ attitude towards QI was evident as nurses reported losing some their learning:

*‘I had to look back over my practicum before we started this chat. Looking back in practice, it’s not something I’ve come across, or think about daily. To get involved, I would need to study again for more knowledge, and then maybe some of this stuff would come back to me.’*

(Interview G)

### 3.3. Influencing Factors

#### 3.3.1. Hierarchy

After qualifying, some of the participants claimed they had no exposure to QI activity. One nurse stated that QI was undertaken by management or staff working towards promotion and often in their own time. A hierarchy often deterred nurses from engaging in QI:

*‘It was mostly higher up that were initiating the changes—one of my colleagues tried quality improvement stuff, but she got a lot of push back by higher up management, so you need to jump these barriers all the time.’*

(Interview H)

This hierarchical exposure to QI was validated as one nurse, now in a senior post, spoke of only just then receiving exposure to the information needed to engage with QI. This nurse reported having full autonomy and greater insight into how the organisation functioned, now that they were more involved and keener to advance their services:

*‘It’s quite good to be accepting of change. In my previous post, I feel like I didn’t have the opportunity for QI, whereas I feel now that I’m in a senior role, I feel like I can take a step back and identify ideas that I want to change and make improvements.’*

(Interview C)

#### 3.3.2. Leadership

The nurses were able to draw upon their QI educational experience as a student to identify factors they believed influenced their current QI engagement. Regardless of being a preceptor or senior staff nurse, those engaged with QI further reported the experience of supportive leadership during their QI Practicums:

*‘I was very lucky, our SCN at the time was just so up for QI and so positive and transparent with data. I felt so supported and felt that I had control over doing QI projects when I became a registered nurse.’*

(Interview D)

While this transformational leadership style influenced nurses’ engagement in QI, others experienced a more laissez-faire leadership style which produced opposite effects. Nurses without greater line-management input perceived that they lacked the permission to initiate change, and without clear direction, nurses did not see the point engaging in QI. One nurse requested that clearer expectations from management around the nurses’ role in QI was needed:

*‘There is a real lack of management expectations—but you need authority from them to go and do QI, you need like a recognised person to say go and do it. If you do it yourself, it’s more difficult.’*

(Interview H)

Despite the leadership style demonstrated by nurse managers, there was a recognition by new nurses that staff in all roles were under constant time pressures, which meant committing to QI was difficult; this included management:

*‘I would like to take forward my QI project, but we are not given the time for that, I’m not blaming my manager, she’s under so much pressure on every part. It doesn’t seem to matter what level of the system that you’re actually at, everybody is struggling to make things their priority.’*

(Interview A)

#### 3.3.3. COVID-19 Pandemic

The COVID-19 pandemic was referred to by many nurses as a reason they did not participate in QI, even where they had retained QI knowledge or experienced supportive leadership. One nurse reported that their QI project involving the interprofessional team was put on hold to deal with the core requirements of working in the pandemic. While this nurse reported getting involved in QI again as services returned to normal, others emphasised that the lasting effects of COVID-19 had been ‘hellish’ and ‘chaotic’. This experience had resulted in burnout for some nurses and hindered their engagement in QI:

*‘The aftermath of COVID was actually worse, the atmosphere is just totally different, and it’s been a really hard year, and I haven’t got a break. Everyone is just done. Even if you see people trying to improve something, a lot of people just aren’t interested.’*

(Interview F)

Burnout from the COVID-19 pandemic was reported to come from caring for sicker patients and managing the ever-changing demands placed upon them throughout this time. As a result, nurses reported having to psychologically prioritise their families over their work and do only what was necessary in practice before going home. However, there was an indication that while COVID-19 no longer posed the same set of challenges, its lasting effects, such as a lack of engagement in QI, had continued:

*‘COVID has a lot to say about forgetting about QI for me. It’s also been a good excuse for people not returning to some key principles that we should be focused on.’*

(Interview A)

#### 3.3.4. Data Access

When nurses were asked if they could access the information needed to identify areas for improvement, some demonstrated negativity towards statistics. For instance, nurses who had progressed into senior positions reported feeling confident when asking for the necessary data, but those remaining at the frontline admitted to not knowing where to retrieve it and associated data with negative performance:

*‘We don’t get like statistics on things as a staff nurse unless it’s something negative. You know, you kind of get your audits for things, where we are told where we need to improve. I wouldn’t know where to start in terms of getting access to other data though.’*

(Interview H)

Some of the participants asserted that despite being qualified nurses, their experience of needing permission to access data through gatekeepers remained similar to their experience as a student during the QI Practicum. Consequently, nurses lacked the necessary information needed to engage in QI and so they did not try. Contrastingly, other nurses retrieved data with ease which was attributed to having login credentials for online health systems where they could explore problems. These nurses were able to demonstrate their retained QI knowledge and positive attitudes round the importance of data:

*‘It wasn’t a problem for us to collect data as registered nurses, our manager was open about it, you know, just being honest, it’s good to have that transparency, we knew exactly where we were. You could actually implement changes because you know the data was accurate. I know others in the hospital struggle, so I don’t know if that’s just because of where we were.’*

(Interview D)

#### 3.3.5. Location

The nurses had developed their QI learning as students during the QI Practicum in the same acute hospital setting. As nurses they were drawing upon their experiences of QI engagement from acute and community settings across different regions in Scotland:

*‘So, when I moved from one health board to another, I was a charge nurse and really keen to implement a lot of changes. But, when I got there, I experienced a lot of barriers and there was more politics in that hospital than in my last one.’*

(Interview D)

The practice setting in which nurses worked was a key influence on QI engagement. Despite displaying positive individual characteristics such as embracing challenges, having an intrinsic value to do better, upholding a passion for nursing, and practicing a growth mindset, if the location did not exude a positive QI culture, engagement was difficult:

*‘Location and the staff you work with has a lot to do with QI—it really depends on where you are, some health boards are very clique and hierarchical.’*

(Interview B)

### 3.4. Transferrable Skills

There was evidence that nurses transferred their QI knowledge and skills inside and outside of the healthcare environment. One nurse who had substantial experience of supporting QI spoke of drawing upon their expertise in a new post. In their annual professional development plan, they had objectives to create a ‘QI link nurse’ role and mentor others:

*‘Just before I went on maternity leave, I had my annual review and I agreed to go back and develop a role in QI, I would love to be able to do that again. I love where I work, and they are big on QI.’*

(Interview D)

Moving beyond the healthcare environment, one nurse described their engagement with their son’s school improvement agenda to enhance the learning experience of young people in primary education. They had transferred QI knowledge and skills learned during their degree to benefit the wider community:

*‘I’m engaging a lot more with the community and have found a lot of purpose improving well-being. QI can be used in all areas, and I’m engaged with the school for example to develop a community garden initiative. For me, I would work in the community for health improvement, but I don’t see a lot of opportunities for that.’*

(Interview A)

Lastly, despite the challenges faced, most nurses agreed that QI was a transferable skill, if nurses remained engaged. They saw the importance of QI as a taught component to continue within degree level nurse education and identified its value in raising awareness of change and ensuring the future generation of nurses continue to improve and grow.

## 4. Discussion

Preregistration nurses in the UK will inevitably encounter QI education within their nursing degree and there has never been a more critical time to have frontline nurses demonstrate competence in healthcare and service improvements. Our study, the first follow-up study of its kind, set out to investigate if registered nurses who completed a practice-based QI project in the final year of their degree remained engaged with QI once qualified. While this generation of new nurses educated in QI can create and foster a culture of improvement in practice, our study which presents a snapshot of this phenomena, suggests that much work is still needed to support this agenda.

NQNs entered the profession with different views on the value of receiving QI training and undertaking a QI project in practice. The unique position that students were exposed to by receiving QI training was emphasised to them regularly during their degree. Some students capitalised on this QI training as an attribute to enhance their employment prospects. However, the analysis showed that some employers and NQNs did not regard QI as an essential skill to attain early in their professional career. This perception may have been influenced at the time by the Flying Start Programme, which new nurses undertook to support their preceptorship year, as it made no reference to QI standards. In 2019, this transition programme was refreshed to reflect the ever-changing needs of the environments in which NQNs work. NQNs are now required to reflect upon and attain evidence linked to quality and service improvement [14]. Therefore, healthcare employers will be compelled to support NQNs and provide opportunities for them to engage in QI activities. This responsibility has implications for practice leaders who will need to reassess their own nursing team’s QI involvement and consequently plan suitable QI-related learning activities for new nurses to evidence their QI competences. Further, as NQN enter the profession and become accountable for their learning, they need to actively seek out QI-related opportunities to become involved. In the past five years, the increased momentum of QI nurse educational research has emerged due to the QI alignment between faculties and professional nursing standards [2,3,4,5,6]. Given this shift, there are now greater opportunities to investigate the views of NQNs in the current context in order to determine what value they place on their QI education and their role within it during their preceptorship year. The requirements of healthcare employers to support NQNs to engage with QI activities will need to be understood to enable effective strategies to be put in place. We recommend that research is prioritised in this area to ensure that we can foster and benefit from these nurses’ QI knowledge.

There is now a professional requirement and desire from practice partners to have new nurses enter the profession with sufficient knowledge of improvement theories and tools [13,35]. The nurses in our study were uniquely positioned to enter the profession with QI training and knowledge. Faculties had selected an experiential learning approach in practice to tutor students about QI due to its ability to enhance QI knowledge [2]. However, the findings showed that educational efforts to develop nurses’ QI knowledge were reversed where a lack of engagement opportunities in QI activity existed. Nurses’ lack of knowledge is one of the most often reported barriers to engagement in QI [25,27,36]. As such, greater alignment between academic and practice partners will be essential to create an infrastructure that builds upon new nurses’ QI capabilities and supports their ongoing professional development [35,37]. Practice education facilitators could be central in this role given their oversight of nurses’ learning needs and their contribution to developing regular practice training updates. Their role in evaluating what current opportunities exist for new nurses to engage in QI could be useful to inform future educational pathways. Fostering engagement in QI activities for new nurses can create future QI leaders and change agents, as was shown in our study through the specialist roles nurses adopted, such as the QI link nurse. Capacity building in this way could be a solution to increase the QI knowledge of future front-line early career nurse managers, of whom only 30% reported feeling prepared to undertake QI [27]. Further, nurse leaders and nurses report wanting to learn together through workshops and drop-in seminars, and seek to partner together to work on QI projects [36]. Learning through QI academies, of which there is evidence in UK health boards, could enable such QI partnerships to be developed [38]. Future research studies should examine the feasibility of new nurses and leaders partnering together in QI activities and determine how QI academies might help in sustaining new nurses’ QI knowledge after they enter the profession.

This study highlighted and found that QI was considered by nurses as a progressive responsibility undertaken as their roles advanced. This view exposed a culture in which QI was not considered an everyday nursing role. Hierarchy in organisations is often reported by nurses as a barrier that prevents them engaging in QI activity [25,36]. Our findings suggest that nurses brought ideas for change forward but did not feel like their managers listened. A potential reason for this lack of consultation may be due to nurse leaders facing their own hierarchical barriers such as disproportional funding for QI projects between disciplines and lack of physician buy-in, which can take several months of persuasion [25,36]. A lack of autonomy and ability to speak up to challenge leaders was discussed by some of the nurses in our study. This skill is necessary to enable nurses to advocate for change and improve patient care and services. Thus, further investigation into the personal attributes of nurses and how they influence QI engagement is needed. Further, future research that investigates how the power dynamics between disciplines impact nurses’ engagement in QI will be necessary to develop strategies that enable nurses at all levels to have a voice which reinforces the values and behaviours that underpin high-quality care.

Our findings showed that where nurse leaders modelled positive QI values and behaviours during nurses’ degree, nurses reported sustained engagement in QI. Leadership has been significantly associated with the success of QI and attributed to high-functioning teams [26]. Nurses believe leaders are highly influential in encouraging engagement in QI where they adopt transformational leadership styles through creating buy-in, supporting a just culture, and working in partnership with more junior staff [36]. In contrast, our study showed that where co-operation between nurses and leaders was based upon a laissez-faire approach to QI activities, it led to unclear expectations about QI roles. This confusion hindered active engagement and mirrors findings found within other studies [39]. Leadership influence in QI appears to begin during students’ degree and has potential to affect engagement in QI once qualified. Therefore, leaders involved with QI will need to assess and reflect upon their own leadership styles when supporting new nurses and students undertaking QI. Students were distributed to one of the many practice settings to undertake their QI project, and thus students’ exposure to a leader who modelled positive QI values and behaviours was serendipitous. Students would benefit from undertaking QI activities across a range of settings to optimise their chances of working in a culture conducive to QI and increase the likelihood of engaging in QI once qualified. These changes would have implications for how QI is taught in educational programmes, because curriculum developers will need to redesign their modular content to include more frequent experiential learning opportunities for students to engage in QI.

During the COVID-19 pandemic, opportunities for rapid improvements and catalysing change efforts were at the forefront of all health professionals’ practice. For the nurses in our study, however, it was a stressful period during which QI activity was postponed and no engagement in QI was reported. These findings should be interpreted with caution though, as nurses referred to COVID-19 being used as a cover-up to justify why engagement in QI had not resumed post-COVID. The emotional stress that nurses faced could have also potentially skewed their memories during such events.

Using data to monitor performance is the cornerstone of improving patient care and services. Student nurses are expected to acquire knowledge of data before qualifying and during their degree; students collected, analysed, and displayed data using run charts for the QI Practicum [13]. Earlier studies report that students have lacked information about where to collect data; perceived data to be tedious, uninteresting, boring, or non-educational and irrelevant; and 60% of student in a survey replied they had not collected any data using fishbone diagrams or process maps during their QI projects [12,18]. If students’ QI learning experiences influence their engagement in QI once qualified, then faculty need to ensure effective QI learning activities around data are developed. The qualified nurses in our study reported only having access to data in QI roles or in senior positions. This outcome aligns to a survey in which nurse leaders were more likely to report access to data as a facilitator of engagement in QI than clinical nurses [40]. However, data must be used to generate open and transparent conversations about improvements between leaders and nurses if a culture of psychological safety is to be developed. Scrutiny of our analysis instead reveals that nurses developed a negative association with data (or statistics) and had a fear of wrongdoing. These findings show that efforts in education and practice should focus on developing positive attitude and beliefs around data if QI engagement is to be increased.

Nurses in our study displayed positive beliefs and attitudes about QI and had a will to improve patient care. Despite these best intentions, they asserted that QI engagement was decided upon by the location in which they worked. This finding is unsurprising given the contextual nature upon which QI depends for success [26]. Location encompasses many interacting contextual factors that influence engagement in QI, such as leadership, mentorship, data, culture, and hierarchy [36,40]. While understanding these facilitators and barriers are useful in figuring out new nurses’ engagement in QI, research should go beyond qualitative and quantitative methods to employ a realist methodology which answers ‘what works, for whom and in what circumstances?’ [41]. Developing rich case studies to explore ‘why’ these facilitators and barriers exist in different contexts will help in recommending future QI engagement strategies for nurses. Phase one of the study revealed that students’ locations presented associated challenges for not engaging with their QI Practicums, namely in regard to familiarity and size of the setting, shift type and patterns, balancing workloads, and the presence of QI expertise. While nurses in our study did not discuss these factors in depth, they could be key to planning effective engagement in QI for new nurses and thus warrant further investigation.

Lastly, a surprising finding relating to the transferability of nurses QI skills was identified. While some nurses transferred their QI knowledge and skills to new nursing roles, others applied them to real-world issues outside of healthcare, such as early years education. These findings coincide with attempts seen to optimise QI education and improve wider societal concerns. In undergraduate medical education, the SusQI education framework links concepts of sustainable healthcare with QI methods [42], and in New Zealand, nurse education aligns cultural agendas to the QI curriculum to improve equity and inclusion of the Māori culture [6]. These findings highlight the potential reach of QI education and the possibility of exploring QI projects being applied to and within different contexts. There are reports of students being more motivated and engaged in QI where there is a shared vision or personal preference in the QI topic being undertaken [43]. While the need for small QI projects in the practice setting still exists, the global demands and responsibilities placed upon us are now greater than ever before. As a result, educational faculties may wish to consider more innovative and engaging pedagogical approaches to teach nurses QI methodology.

## 5. Study Limitations

While this study has generated important findings about nurses’ engagement with QI following QI training within their degree, the sample is limited to one university programme in central Scotland, and the findings should be seen within this context. Despite the small sample allowing us to explore what supports or impedes QI practice post-registration, our findings cannot be generalised to other fields of nursing such as mental health, children, or learning disability due to the different contexts in which nurses practice. A large-scale survey would be needed to determine how representative the findings are in Scotland. In QI research, it is understood that while an intervention works in one setting, it may not work in another due to the complexity of the inter-related contextual factors present. Similar can be said about our own follow-up findings; however, because our findings mirror those established in the wider QI education [2], they serve as a starting point to further explore the factors influencing new nurses’ engagement in QI.

Future research may wish to address a key limitation of this study. Firstly, an extended period occurred between students qualifying and follow-up interviews being conducted. Recruitment was planned for the first quarter of 2020 but postponed due to non-COVID-19-related research being temporarily ceased. This delay may have resulted in recruiting only 10 participants from the original sample of 30, due to invalid contact details, student nurses no longer seeing the relevance of taking part in the research or the attrition of nurses leaving the profession following the COVID-19 pandemic [44]. While there was also a potential for selection bias, such as nurses who were more involved in QI agreeing to be interviewed, our participants included nurses not involved in QI and those no longer in the profession. Consistent with other qualitative studies, our sample was sufficient within the parameters of our defined aims, and we reached data saturation before the end of the data collection period [45]. Secondly, the extended period potentially affected the reliability of nurses’ recall during the earlier years, which included transitioning to a newly qualified nurse and the preceptorship year. Thus, the cognisance of the accuracy of nurses’ lived experiences should be accounted for when interpreting the findings. For instance, it may have resulted in events being over- or under-reported or heightened states during the pandemic leading to distorted nurses’ memories. Triangulation during data collection should be considered in future research follow-up studies to mitigate recall bias. This could be achieved through regular entries about nurses’ QI engagement in a participant reflective diary. This could have potentially allowed our findings to be explored in greater depth and strengthened our findings. Given the similarity and consistency of participants’ interview responses in line with the wider literature, we are confident the findings present a trustworthy and valid depiction of how nurses engage with QI in practice following their degree education.

## 6. Conclusions

Qualified nurses have the potential to become agents of future change and sustain their knowledge and engagement in QI following the completion of a QI project during their degree. However, the juxtaposition in supporting new nurses to become engaged in QI activity will need to be carefully considered between educational institutions and practice partners. To foster QI engagement in new nurses, multiple QI learning opportunities made available during pre-registration degree programmes should be developed and continued through ongoing professional development and in collaboration with nurse leaders in practice, who can reflect upon their own leadership styles and organisational culture. The role that practice education facilitators play in identifying suitable QI learning opportunities could enable more innovative pedagogical approaches in QI to be developed within nursing programmes. Lastly, the greater exploration of the influencing factors affecting new nurses’ engagement in QI could be enhanced by investigating a larger sample using realist evaluation methodology.

## Figures and Tables

**Table 1:** The QI Practicum curriculum content [8].

Semester	IHI Open School Courses	Teaching
1.		Person/patient-centred care Introduction to the concept of quality in healthcare Example of quality initiatives in action—Scottish Patient Safety Programme.
2.	QI101 Fundamentals of Improvement PS100 Introduction to Patient Safety PS101 Fundamentals of Patient Safety	QI Questions in MCQ exam Evidence-based learning
3.	PS102 Human Factors and Safety PS103 Teamwork and Communication Q102 The Model for Improvement: Your Engine for Change	Evidence-informed practice Quality improvement model
4.	QI103 Measuring for Improvement QI104 Putting it All Together: How QI Works in Real Health Care Settings	Improvement and Safety QI questions in MCQ exam Practice-based assignment—Care Partnerships, Care Study
5.	QI106 Level 100 Tools	Tools for quality improvement
6.	PS 104 Root Cause and System Analysis PS105 Communicating with patients after Adverse Event	Decision-making Evidence for practice Resources for Practicum online
7.	PS106 Introduction to the Culture of Safety L101 So you want to be a Leader in Health Care	Podcasts 2 Introduction to Practicum Practicum: essential skills workshop Preparing to work at SCQF level 10 Practicum assignment Q&A Resilience workshop
8.		Collaborative improvement project (Practicum) Practice events Online/email/telephone/interview/workshop support Reading week

**Table 2:** Characteristics of study sample.

	Gender	Age	Current Designation/Employment	Engagement in QI Once Qualified	QI Practicum Location as a Student Nurse	Contextual Factors Influencing Engagement in QI Practicum
Participant A	Female	47	Staff Nurse Acute Medicine	Reported involvement in QI initiatives	Medicine	Confidence to articulate ideas and persistence to obtain early manager buy-in facilitated engagement.
Participant B	Female	29	Senior Staff Nurse Community	Reported involvement in QI initiatives	Medicine	Supportive QI culture and knowledgeable mentors encouraged and facilitated QI engagement.
Participant C	Male	32	Senior Staff Nurse Acute Medicine	Reported involvement in QI initiatives	Surgical	Knowledgeable senior charge and positive change culture permitted autonomy to engage in QI.
Participant D	Female	36	Staff Nurse Community	Reported involvement in QI initiatives and application of QI methods/tools	Surgical	Knowledgeable senior charge and positive change culture permitted autonomy to engage in QI.
Participant E	Female	47	Senior Staff Nurse Community	Reported involvement in QI initiatives	Rehabilitation	Jaded mentors’ attitudes and senior leadership changes reduced ability to engage in QI activity.
Participant F	Female	32	Staff Nurse Acute Medicine	No involvement	Medicine	Hierarchy meant that staff did not perceive students’ QI projects as important; in turn, this reduced student’s engagement.
Participant G	Female	29	Staff Nurse Acute Medicine	No involvement	Telephone Support	Too many student projects going on simultaneously which reduced support to engage in QI.
Participant H	Male	34	Staff Nurse Acute Medicine	No involvement	Medicine	Active QI culture made it difficult to find a QI project to engage in as ‘everything was already being done’.
Participant I	Female	32	Staff Nurse Acute Medicine	No involvement	Medicine	Familiarity of placement and positive growth mindset enhanced QI engagement.
Participant J	Female	35	Former Staff Nurse Left the Profession	No involvement	Medicine	Practicalities of large complex environment posed challenges to timely engagement.

## Data Availability

Ethics permission does not allow data sharing of interview data.

## Supplementary Materials

The following supporting information can be downloaded at: https://www.mdpi.com/article/10.3390/nursrep14040218/s1. File S1: Interview Questions Follow Up Phase; File S2: Phase 1 Ethnographic Coding.

## Public Involvement Statement

No public involvement in any aspect of this research.

## Guidelines and Standards Statement

This manuscript was drafted against the COREQ 32-item Checklist for Qualitative Research [31].
