# Supplementary material for: Experiences of Newly Qualified Nurses’ Engagement with Quality Improvement in Practice: A Qualitative Follow-Up Study
Source: Nurs Rep. 2024 Oct 14;14(4):2990–3006. doi: 10.3390/nursrep14040218 (PMC11503377; doi:10.3390/nursrep14040218)
Supplement: Supplementary file 1 [file nursrep-14-00218-s001.zip › File S1 Interview Questions Follow Up Phase.pdf]

Lorraine Armstrong
PhD Candidate
Lecturer in Nursing
University of Stirling

Follow-Up Study Topic Guide for interviewing nurses who participated in the Quality Improvement Practicum

General – 10 minutes

- Thanks and welcome
- Nature of interview
- There are no right or wrong answers
- Audio recording process
- Gain verbal consent
- Explain colleague viewing
- Re-iterate participant can stop interview at any time
- Remind nurse about their previous reported experience of QI Practicum
- Check that this information correct – and update if necessary
- Questions or concerns?
- *Start Recording*

CONSENT TO READING INFORMATION LEAFLET AND TO PARTICIPATE IN STUDY

Contextual information

1. Where do you work now?
2. What is role as a nurse now?
3. Have you been in different roles in the past 8 years?

Transition to Newly Qualified Nurse

4. In hindsight, what benefits (or not) did undertaking QI have on your ability to get a job?
5. Have employers ever asked you about your QI expertise?
6. Have you ever included it in your CV or job application?
7. Have you included any QI reflections/PDP within your Revalidation?

Current engagement in QI

8. What further training or learning around QI have you done since your BSc?
9. What knowledge do you believe that you have retained about QI since then?
10. How would you regard your own QI expertise?
11. Since graduating have you ever had the opportunity to apply your knowledge in practice?
12. What QI work is ongoing in your own environment?
13. How would you describe the QI culture where you work?
14. How would you describe your level of engagement with QI now?
15. Now that you are experienced, what factors in your ward influence engagement with QI?
16. Do you/or would you feel confident supporting others in doing QI? How do you do this?

Drawing upon ethnography experiences

17. Lack of autonomy to do QI was a factor as a student nurse, how do you perceive your level of autonomy to influence your engagement with QI now you are experienced?
18. Students' access to data was limited for undertaking QI, what impact does your understanding of data or access to data have now on doing QI?
19. Student determinants played a key role in students engaging in QI, what do you think are the key attributes required to do QI now as a registered nurse?
20. Often students identified a fear culture in highlighting wrongdoing when selecting QI work? What is your experience of this type of culture now in the wards as a registrant?
21. The study identified a new culture of nurses knowledgeable in QI – do you think the NQN workforce is beginning to impact the overall knowledge of QI in practice?

Attitudes and beliefs about QI

22. What are your beliefs about the nurse's role in doing QI?
23. What value do believe exists in pre-registration nurses undertaking QI in practice?
24. Have you supported any students who are conducting QI in practice or doing an assignment?
25. Was your own experience useful in supporting these students?
26. Would you consider a job in QI?
27. What would influence you to become more involved in QI?
28. Would you recommend QI for pre-registration nurse now?
